# Batch and semi-continuous fermentation with *Parageobacillus thermoglucosidasius* DSM 6285 for H_2_ production

**DOI:** 10.1186/s13068-024-02597-z

**Published:** 2025-01-09

**Authors:** Magda S. Ardila, Habibu Aliyu, Pieter de Maayer, Anke Neumann

**Affiliations:** 1 Section II: Electrobiotechnology, Institute of Process Engineering in Life Science, Karlsruhe Institute of Technology, 76131 Karlsruhe, Germany; 2 Section V: Biotechnology and Microbial Genetics, Institute for Biological Interfaces, Karlsruhe Institute of Technology, 76131 Karlsruhe, Germany; 3 School of Molecular & Cell Biology, Faculty of Science, University of the Witwatersrand, Johannesburg, 2000 South Africa

**Keywords:** *Parageobacillus thermoglucosidasius*, Hydrogen, Water gas shift reaction, Carbon monoxide dehydrogenase, Semi-continuous fermentation

## Abstract

**Background:**

*Parageobacillus thermoglucosidasius* is a facultatively anaerobic thermophile that is able to produce hydrogen (H_2_) gas from the oxidation of carbon monoxide through the water–gas shift reaction when grown under anaerobic conditions. The water–gas shift (WGS) reaction is driven by a carbon monoxide dehydrogenase–hydrogenase enzyme complex. Previous experiments exploring hydrogenogenesis with *P. thermoglucosidasius* have relied on batch fermentations comprising defined media compositions and gas atmospheres. This study evaluated the effects of a semi-continuous feeding strategy on hydrogenogenesis.

**Results:**

A batch and two semi-continuous fermentations, with feeding of the latter fresh media (with glucose) in either 24 h or 48 h intervals were undertaken and H_2_ production, carbon monoxide dehydrogenase (CODH) activity, and metabolite consumption/production were monitored throughout. Maximum H_2_ production rates (HPR) of 0.14 and 0.3 mmol min^−1^, were observed for the batch and the semi-continuous fermentations, respectively. Daily feeding attained stable H_2_ production for 7 days, while feeding every 48 h resulted in high variations in H_2_ production. CODH enzyme activity correlated with H_2_ production, with a maximum of 1651 U mL^−1^ on day 14 with the 48 h feeding strategy, while CODH activity remained relatively constant throughout the fermentation process with the 24 h feeding strategy.

**Conclusions:**

The results emphasize the significance of a semi-continuous glucose-containing feed for attaining stable hydrogen production with *P. thermoglucosidasius*. The semi-continuous fermentations achieved a 46% higher HPR than the batch fermentation. The higher HPRs achieved with both semi-continuous fermentations imply that this approach could enhance the biohydrogen platform. However, optimizing the feeding interval is pivotal to ensuring stable hydrogen production.

**Supplementary Information:**

The online version contains supplementary material available at 10.1186/s13068-024-02597-z.

## Background

Currently, there is an extensive drive for the development of carbon–neutral technologies for energy production, mitigating the negative environmental effects and overreliance on finite fossil fuel reserves [1]. Hydrogen (H_2_) has emerged as an attractive and versatile energy carrier with a high energetic yield, producing only water during combustion [2–5]. Given the high costs and non-carbon–neutral nature of current industrial H_2_ production strategies, increasing research has focused on the development of biological means for production [1]. Explored approaches include direct and indirect bio-photolysis, microbial electrolysis cells, and photo- and dark fermentations [5, 6]. More recently, research has focused on hydrogenogenic carboxydotrophic microorganisms that can produce H_2_ using carbon monoxide (CO) as an energy and/or carbon source [7, 8]. These make use of two metalloenzymes namely a nickel-containing carbon monoxide dehydrogenase (NiFe–CODH) and an energy-conserving NiFe-hydrogenase to catalyze the water–gas shift (WGS) reaction, where CO reacts with water to produce hydrogen and carbon dioxide (CO_2_) [7, 9–12]. CO is an inexpensive compound, present not only in syngas but in other waste gases, and hence, using this substrate for biohydrogen production has the added benefit of reducing carbon emissions [9].

*Parageobacillus thermoglucosidasius* is a thermophilic, Gram positive, rod shaped bacterium in the family Anoxybacillaceae and the phylum Bacillota, which has previously been shown to produce H_2_ gas from CO using the WGS reaction, using a unique carbon monoxide dehydrogenase (CODH) hydrogenase enzyme complex [13–16]. Unlike most other hydrogenogenic carboxydotrophs, which are strict anaerobes, this taxon is a facultative anaerobe, that can grow in the presence of atmospheres containing air and CO, but then shifts to the WGS reaction once oxygen has been consumed [13, 16]. A lag phase between O_2_ depletion and the beginning of H_2_ production has been described, and different parameters were optimized to reduce this lag phase in batch experiments with *P. thermoglucosidasius* DSM 6285 [14].

This study aimed to further increase H_2_ yield and reduce the lag phase before H_2_ production with *P. thermoglucosidasius* DSM 6285. To achieve this, two-phase semi-continuous fermentations containing an aerobic and anaerobic step were performed in 2.5 L stirred tank reactors and compared to a batch fermentation strategy.

## Results

### The effects of semi-continuous fermentation on hydrogen production rates and yield

**Fig. 1:**
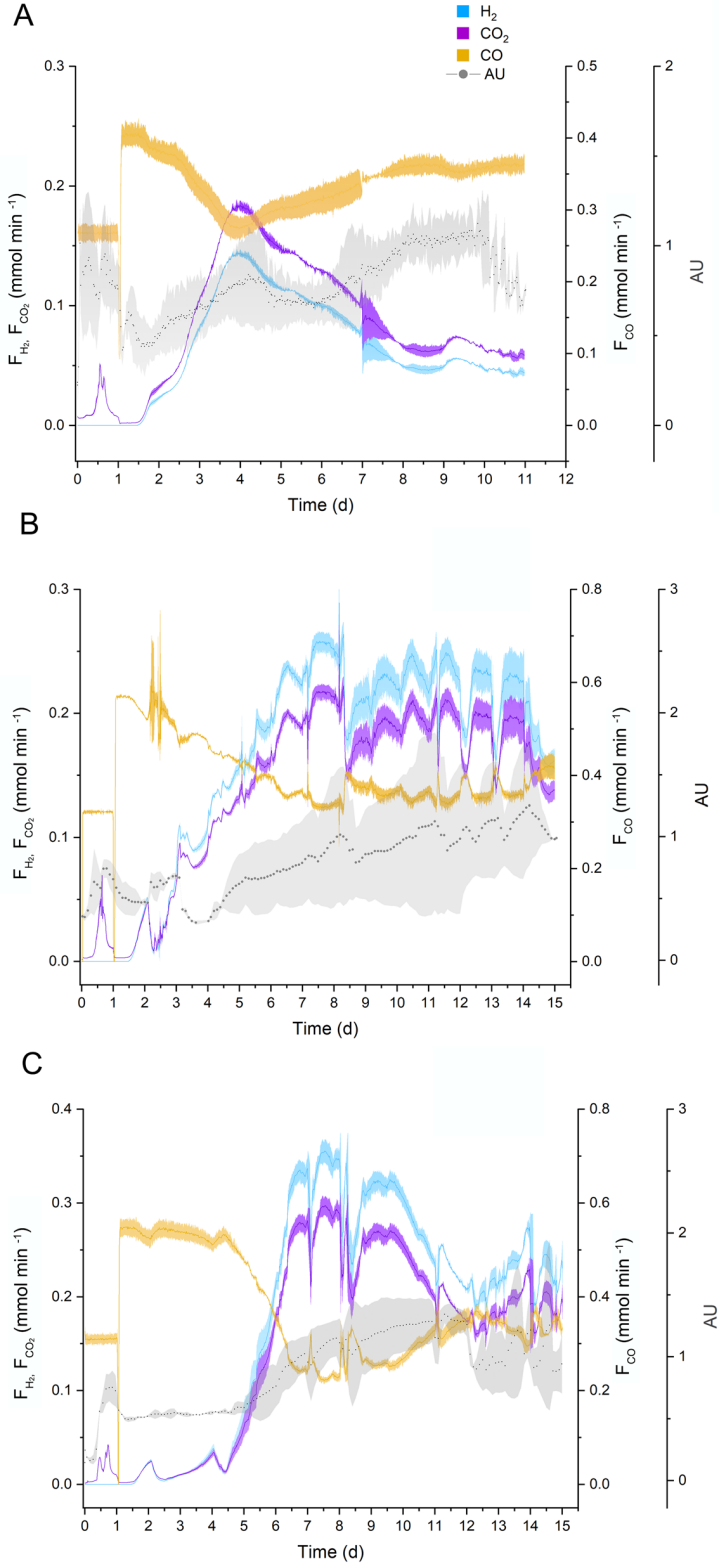
Fermentations in mASM medium. *P. thermoglucosidasius* DSM 6285. The H_2_ and CO_2_ production rate (mmol min^−1^) and the CO consumption rate (mmol min^−1^) with the standard deviation indicated by the colored regions. The biomass, measured as absorbance units (AU), is depicted as a grey line. The aerobic phase ends after 24 h with gas exchange. **a** Batch fermentation over 11 days. **b** Semi-continuous fermentation with feeding every 24 h, starting from day 2. **c** Semi-continuous fermentation with feeding every 48 h starting from day 2

Batch fermentations were first performed, where *P. thermoglucosidasius* DSM 6285 was grown in duplicate STR bioreactors containing defined modified Ammonium Sulphate Medium (mASM) [17] and fed with a continuous flow of CO. Consumption of CO started on day 2 concomitant with the production of H_2_ in the reactors. A maximum hydrogen production rate (HPR) of 0.144 ± 0.002 mmol min^−1^ was observed on the fourth day of fermentation (Fig. 1A), followed by decreased H_2_ production throughout the remainder of the fermentation, plausibly due to the growth-limiting conditions imposed by the complete consumption of glucose and build-up of toxic metabolites. The semi-continuous approach, undertaken in the same STR reactors, involved the feeding of the bioreactors with 60 mL d^−1^ mASM medium containing 5.5 mM glucose every 24 or 48 h. Similar to the batch fermentation, the gas flow was kept constant throughout the fermentation.

The 24-h interval feeding proportionated the first stable hydrogen production after 6 days of fermentation. Hydrogen production started from the second day, increasing to 0.26 ± 0.01 mmol min^−1^ by day 7, a volumetric HPR of 7.98 L H_2_ L^−1^ d^−1^, with a hydrogen yield of 0.22 mmol H_2_/mmol CO of a flow rate of 0.71 mmol CO min^−1^. CO consumption increased from the second until seventh day, and remained stable throughout the rest of the fermentation (Fig. 1B). With the 48-h interval feeding, H_2_ production was delayed to day 7 of the fermentation, when it increased to an HPR of 0.32 ± 0.01 mmol min ^−1^ and a volumetric HPR 11.91 L H_2_ L^−1^ d^−1^ (Fig. 1C). Subsequently, instability in the H_2_ production profile was observed, decreasing to 0.23 ± 0.02 mmol min ^−1^ on day 8, followed by recovery 0.32 ± 0.01 mmol min ^−1^ by day 9, before decreasing to a minimum HPR of 0.188 ± 0.01 mmol min ^−1^ (7.65 L H_2_ L^−1^ d^−1^) by day 13. This was likely due to the formation of foam, which caused changes in the CO uptake rate from day 11.

### Relationship between HPR and CODH activity

**Fig. 2:**
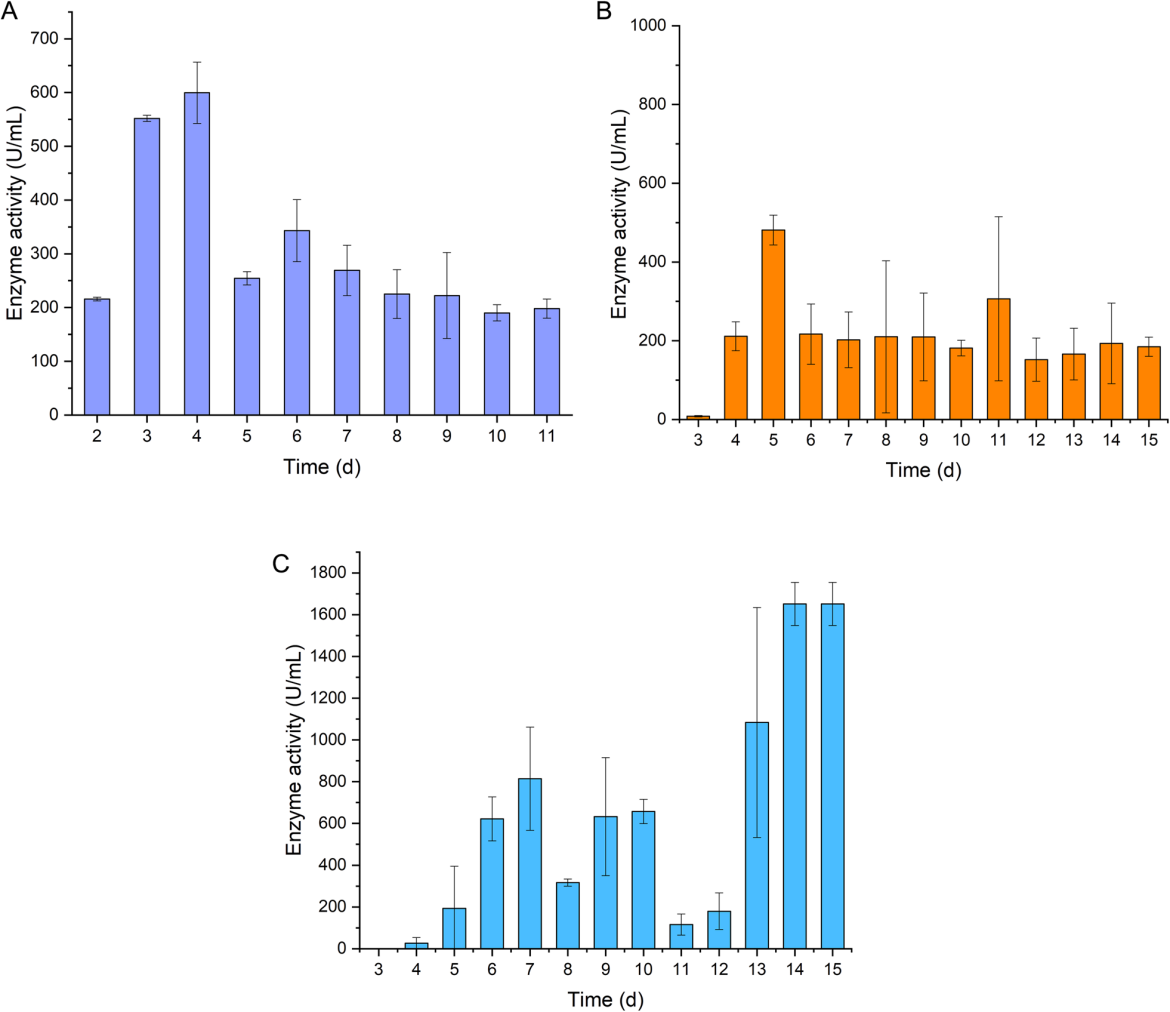
CODH enzyme activity for **a** batch fermentation, **b** feeding every 24 h, and **c** 48 h. The enzyme activity for the semi-continuous fermentations was measured from the third day of fermentation

To determine whether H_2_ production and enzyme activity were correlated, CODH activity was measured for the batch and semi-continuous fermentations using previously described spectrophotometric assays based on methyl viologen reduction [18, 19]. For the batch fermentation, the activity was measured from the second day of fermentation, after the gas exchange, as H_2_ production started. The maximum activity, 599 U mL^−1^, was achieved on the fourth day of fermentation and corresponded to the peak of H_2_ production with an HPR of 0.144 mmol min^−1^ and a volumetric HPR of 4.62 L H_2_ L^−1^ d^−1^ (Fig. 2A). From day 5 to day 11, a reduction of the CODH activity was observed, in parallel with the drop on HPR during batch fermentation.

CODH activity was lower than 10 U mL^−1^ for both 24-h and 48-h interval fed fermentations on day 3, but increased substantively throughout the remainder of the fermentations. The activity was measured for both intervals from day 3 of the fermentations until day 14. Overall, CODH activity was higher with the 48-h interval feeding, and reaching a maximum activity of 1651 U mL^−1^ on day 14 (Fig. 2C). The lag phase prior to H_2_ production was longer with the 48-h interval feeding and this correlates with a lag in CODH activity. The CODH activity also remained more stable in the semi-continuous fermentation with 24-h feeding interval, at around 200 U mL^−1^ from days 6 to 10, which was also a period of stable H_2_ production. By contrast with the 48-h interval feeding, peaks and troughs in H_2_ production were observed, which can also be correlated to peaks and troughs in the WGS enzymatic machinery. For example, a low HPR (0.287 mmol min^−1^) on day 8 of the fermentation is associated with a low CODH activity of 316 U mL^−1^. It should be noted that for each feeding interval, at some sampling points, extensive differences in CODH activity were noted between the duplicate bioreactors for each feeding strategy, which could, in the future, be addressed by performing additional sampling. When comparing the batch with the semi-continuous fermentations, CODH activity is constant after the highest point of H_2_ production during the batch and the 24-h interval fermentations, suggesting that the enzyme is active throughout fermentation.

### Metabolites analysis during the fermentations

**Fig. 3:**
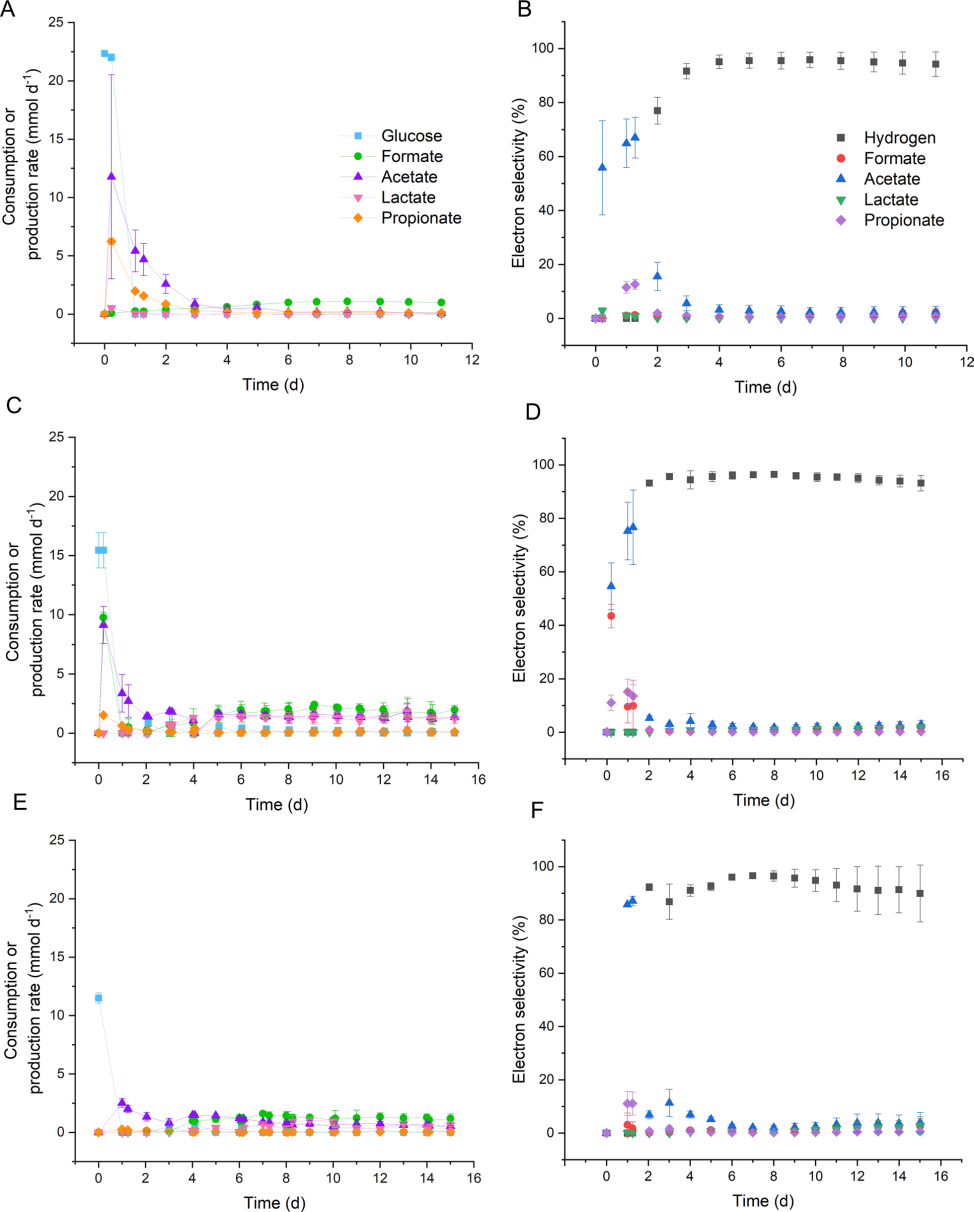
Metabolites during the fermentations in mASM medium. *P. thermoglucosidasius* DSM 6285 was cultivated at 55°C, 500 rpm, and pH 6.8. The production or consumption rate (only for glucose) of the metabolites is the average of two bioreactors, with error bars indicating standard deviation. Metabolite production/consumption rates for the **a** batch fermentation, **c** 24-h feeding and **e** 48-h feeding. Electron selectivity for the **b** batch, **d** 24-h feeding and **f** 48-h feeding semi-continuous fermentations, respectively

The metabolic profiles of *P. thermoglucosidasius* DSM 6285 during the batch and both 24-h and 48-h interval feeding semi-continuous fermentations were evaluated using High Performance Liquid Chromatography (HPLC) analysis. Under aerobic conditions, *P. thermoglucosidasius* produces acetate, lactate, succinate, glyoxylate and a spectrum of other metabolites, while when growing anaerobically in a CO-containing atmosphere, more extensive acetate production has been reported [17]. As observed in Fig. 3, four key metabolites were observed in the batch and semi-continuous fermentations, formate, acetate, lactate, and propionate, while other metabolites such as butyrate, glyoxylate, ethanol, valerate, and citrate were below the detection limit of the HPLC method (< 0.1 g L^−1^). After glucose and acetate depletion in the batch fermentation, at day 4, is possible that citrate is consumed, which could explain the increase in growth from days 7 to 10. However, this has not been described before. It is also known that *P. thermoglucosidasius* has genes linked to the citrate cycle [13].

Formate was the primary metabolic product observed in both batch and semi-continuous fermentations. The second most prevalent metabolite, acetate was produced primarily in line with glucose consumption in the batch fermentation and subsequently tapered off, while in the semi-continuous fermentation it continued to increase largely in parallel with glucose addition. Key differences were observed between the batch and semi-continuous fermentations in terms of propionate and lactate production. Limited propionate production was observed in the semi-continuous fermentations with 48-h feeding interval. Similarly, while negligible lactate production was observed in the batch fermentations, large amounts of lactate up to 18.3 mmol and 9.5 mmol by 15 days were produced in both semi-continuous fermentations. This could be due to the additional glucose in the media that routes the metabolism to organic acid production, and the consumption of acids after each feeding reduces accumulation that can be detrimental for the microorganism growth.

Formate production rate was constant throughout the fermentation with the 24-h feeding strategy, reaching a maximum of 2.41 ± 0.16 mmol d^−1^. Acetate reached a maximum production rate of 9.15 ± 0.55 mmol d^−1^ by 0.2 days and 1.36 ± 0.03 mmol d^−1^ by the end of the fermentation. Lactate had a maximum production rate of 1.82 ± 0.23 mmol d^−1^ on day 13 and later reduced to 1.22 ± 0.41 mmol d^−1^ by day 15. Propionate had a maximum production rate of 1.52 ± 0.15 mmol d^−1^ on day 1, but throughout the fermentation, the production rate was lower than 1 mmol d^−1^. Glucose was consumed by the first day of fermentation, and it increased after the glucose-containing feeding and depleted by the next day.

The 48-h feeding strategy also had formate as the main metabolic product, with production rate 1.61 ± 0.18 mmol d^−1^ by day 7. Acetate had a maximum production rate of 2.51 ± 0.37 mmol d^−1^ on day 1. Lactate started to be produced from day 4 and had a maximum production rate of 1.07 ± 0.17 mmol d^−1^. The propionate production rate was below 0.5 mmol d^−1^ throughout the entire fermentation. Glucose consumption was observed 1 day after the feeding, as observed with the 24-h feeding strategy.

The production of metabolites is significantly shifted to more reduced products, such as H_2_. This is also shown in the electron selectivity, as at the beginning of the fermentations, the electrons coming from glucose were routed into acetate. As soon as the gas exchange was performed on the first day of fermentation, most of the electrons coming from CO and glucose shifted primarily towards H_2_ production.

## Discussion

Continuous fermentation has several advantages over batch fermentation, which is often hampered by substrate limitation and product inhibition, shorter microbial exponential growth phase, and the additional time and cost of cleaning and sterilization of the reactor, ultimately leading to lower productivity [20]. Here we employed both batch and semi-continuous fermentations to evaluate their effect on WGS-driven hydrogenogenesis by the facultative anaerobic thermophile *P. thermoglucosidasius*. Furthermore, two distinct feeding intervals, 24 hourly and 48 hourly, were evaluated for the semi-continuous mode.

In our study, the semi-continuous mode with feeding every 24 h increased H_2_ yield by 18% compared with batch fermentation, and the process could be extended to a longer period. Hydrogen yields declined after 4 days in the batch fermentation, while sustained H_2_ production was achieved over the evaluated period of 15 days with the 24-h interval feeding semi-continuous fermentations. Similarly maintained H_2_ production has been observed in experiments with a dark fermentative microbial consortium grown with cheese whey permeate that was replaced 12 and 24 hourly [21].

Foam formation hampered the 48-h feeding interval semi-continuous fermentations, where the foam can affect the gas transfer to the liquid phase, creating a barrier in the headspace [22]. This precluded meaningful comparison of the effect of different feeding intervals on *P. thermoglucosidasius* hydrogenogenesis. Although there are many strategies to minimize foam, such as defoaming agents and mechanical disruption, the constant change of the medium in fed-batch fermentations is also an alternative [23], as observed with the 24-h feeding interval employed in the study.

While the 24-h feeding interval semi-continuous fermentation facilitated stable hydrogen production for more than 7 days, variations were observed between duplicate bioreactors with both feeding intervals. A broad range of operating parameters, such as gas composition, inoculum size, temperature, pH, stirring rate, and media composition [14], can affect H_2_ production. As such, additional bioreactor and inoculum parameters will need to be evaluated to optimize the semi-continuous fermentation and its reproducibility.

The semi-continuous supplementation with glucose affected the fermentative metabolism of *P. thermoglucosidasius* in several ways. Primarily, in the semi-continuous fermentations substantive acetate and lactate were produced in line with glucose consumption, while these were negligible byproducts of the batch fermentation. Acetate was consumed, once glucose was depleted during the aerobic phase via the presumably TCA cycle, also described by [17]. Once the gas exchange step reduces oxygen of the gas phase, CO consumption through the WGS reaction commences and H_2_ production starts; however, as glucose is added to the feeding, there is a flux of electrons to the production of organic acids mainly acetate, formate, and lactate from pyruvate following glucose metabolism pathways [17]. Under anaerobic metabolism, glucose is oxidized into two molecules of pyruvate, while NAD^+^ is reduced to NADH, afterwards, pyruvate is converted into acetate, and NAD^+^ is regenerated, maintaining glucose oxidation [24]. In our work, all fermentations produced acetate, formate, and lactate suggesting this metabolic route. In addition to glucose, or lack thereof, amino acids in the ASM media such as ʟ-alanine and ʟ-cysteine could be metabolized into pyruvate generating NADH [25].

*P. thermoglucosidasius* DSM 6285 can produce H_2_ via the WGS reaction, catalyzed by the CODH [14]. CODH is sensitive to oxygen at the protein levels [26]. The regulation of the CODH genes is still unclear [13]. The presence of two uptake hydrogenases and one H_2_-evolving hydrogenase in *P. thermoglucosidasius* makes this microorganism distinctive from others, even more so that it has an anaerobic CODH [27]. Spectrophotometric assays based on methyl viologen reduction were used to evaluate CODH activity as proxy for hydrogenase activity in the batch and semi-continuous fermentations [19, 28, 29].

We observed that the CODH activity was constant in both batch and semi-continuous fermentations after reaching the highest H_2_ production. When comparing the feeding intervals, it was noticed that during the 24 h, CODH activity remained stable alongside a period of stability in H_2_ production, while when evaluating the enzymatic activity with the 48-h interval feeding, the overall instability of the H_2_ production reflected the observed variability in the spectrophotometric assays. Nevertheless, certain drawbacks must be taken into consideration. This is evidenced by the differences in CODH activity between the duplicate reactors at several time points, due to the sensitivity of the CODH activity assay to oxygen [26]. Although methyl viologen acts as an artificial electron donor and shares similarities with the natural electron donor, it is not identical [30]. In addition, variations in reaction conditions and redox potential have a pronounced effect on the reliability and reproducibility of the assay [31, 32].

In conclusion, enhanced HPR and extended operational stability were attained using semi-continuous fermentation with a 24-h feeding strategy. Persistent challenges with the 48-h feeding interval, however, underscore the need for further optimization of the semi-continuous fermentation approach for WGS-driven hydrogenogenesis with *P. thermoglucosidasius.* Further evaluation of different feeding rates, environmental conditions and better control and monitoring of foam formation, can mitigate operational challenges to the semi-continuous strategy and concomitantly improve hydrogen productivity over extended fermentation periods.

## Methods

### Microorganism and media

*Parageobacillus thermoglucosidasius* DSM 6285 was obtained from the Deutsche Sammlung von Mikroorganismen und Zellkulturen (DSMZ, Braunschweig, Germany) and was conserved in glycerol (80%) stocks at − 80 °C. Routine cultivation of *P. thermoglucosidasius* DSM 6285 was performed in mLB (modified Luria–Bertani) medium containing tryptone (10 g/L), yeast extract (5 g/L), NaCl (5 g/L), 1.25 mL/L NaOH (10 g/L) and 1 mL/L of each of the filter-sterilized stock solutions 1.05 M nitrilotriacetic acid, 0.59 M MgSO_4_·7H_2_O, 0.91 M CaCl_2_·2H_2_O and 0.04 M FeSO_4_·7H_2_O, as described before [14]. The reactor medium used was mASM (modified ammonium sulfate) medium, containing 8.7 mM citric acid, 20.2 mM MgSO4, 10 mM K_2_SO_4_, 22.6 mM NaH_2_PO_4_, 0.8 mM CaCl_2_, 25 mM (NH4)_2_SO_4_, 4.162 mM glucose, and the trace elements in the final concentration of 0.012 mM H_2_SO_4_, 0.002 mM CuSO_4_, 0.004 mM CoSO_4_, 0.010 mM ZnSO_4_, 0.046 mM FeSO_4_, 0.006 mM NiSO_4_, 0.018 mM MnSO_4_ and 0.002 mM H_3_BO_3_ [13, 17].

### Inoculum preparation

A volume of 300 µL of glycerol stock was added to 200 mL of mLB medium in 500 mL shake flasks and grown under aerobic conditions at 60 °C and rotation at 120 rpm in an Infors Thermotron (Infors Thermotron, Infors AG, Bottmingen, Switzerland). The shake flask were closed using stoppers and covered with aluminum foil, which helped to reduce evaporation of the media, in addition to the short incubation time. After 14 h, a calculated volume of the inoculum was added to the reactors to achieve an initial absorbance (OD_600_) of 0.1 for a total volume of 1 L.

### Experimental setup

#### Batch fermentation

Batch fermentation was performed in two bioreactors of 2.5 L capacity (Minifors, Infors AG, Bottmingen, Schweiz) with a 1 L working volume for 11 days. The stirrer speed was set to 500 rpm, temperature to 55 °C and pH to 6.8; the pH was routinely monitored using a pH probe (Easyferm plus, Hamilton, Switzerland) and controlled with NaOH (1 M) and H_2_SO_4_ (1 M) via a peristaltic pump connected to the reactor system. The fermentation was performed in two phases. First, fermentation was performed using a continuous flow rate of 4.46 mmol min^−1^ of air and CO for 24 h. This was performed by sparging the liquid through the microsparger present in the reactor. Subsequently, the headspace gas was exchanged with 3.57 mmol min^−1^ of a mixture of 20% CO and 80% nitrogen. During the gas exchange, the headspace of the reactor was flushed for 10 min with N_2_ at a flow rate of 0.1 L min ^−1^ to reduce the oxygen content in the reactor.

### Semi-continuous fermentation

The semi-continuous fermentation was performed following the setup described for the batch fermentation. During each feed, 60 mL of fresh mASM medium was fed while removing the same volume of media at a rate of 1 mL min ^−1^. For each reactor, a liquid sample (1 mL) was collected in triplicate at each sampling time to evaluate growth by absorbance and the metabolic profile through an HPLC analysis. An additional sample of 5 mL was withdrawn to evaluate the CODH activity.

### Analytical methods

The gas composition of the bioreactor head space was evaluated online with a 3000 Micro GC gas analyzer (Inficon, Switzerland) connected with Molsieve columns and PLOT Q for data acquisition. The absorbance (OD_600_) was measured using the Ultrospec 1100 pro spectrophotometer (Amersham Biosciences, Uppsala, Sweden). The samples for HPLC analysis were diluted 1:5 with ddH_2_O; prior to storage at −20 °C. After thawing for the analysis, samples were transferred into 1.5 mL HPLC vials. A 5 mM H_2_SO_4_ solution was used as the mobile phase, 55 °C was selected as the column temperature, and samples were analyzed at a flow rate of 0.6 mL min ^−1^ flow rate for 40 min per sample and injection volume of 10µL. HPLC was undertaken using the Agilent 1100 series HPLC system (Agilent Technologies, Waldbronn, Germany) was used, equipped with a wavelength detector and refractive index detector with a 50 mm long pre-column (model Rezex ROA–Organic Acid H^+^ 8% Guard Column) and a 300 mm long separation column (model Rezex ROA–Organic Acid H^+^ 8%). Data acquisition and analysis were performed with the software Chemstation (Agilent Technologies). The gas composition was calculated according to the ideal gas law, as described before [27]. The electron selectivity was used to show the electron flux in the process. This was calculated from the electron mole (e^−^ mol) of each compound. Additional information on the calculations can be found in Additional File 1.

### CODH enzyme assay

CODH enzyme activity was evaluated using a colorimetric enzyme assay based on reducing methyl viologen (MV) under anaerobic conditions [18, 19]. The reduction of 10 mM MV^2+^ dichloride solution in 50 mM Tris–HCl to MV^+^ along the oxidation of CO to CO_2_ results in the change of color of the solution to dark violet color (Eq. 1). One unit of CO dehydrogenase activity is defined under these conditions as that amount required to catalyze the reduction of 1 µmol min ^−1^ methyl viologen:

1 $$\text{CO}+ {\text{H}}_{2}\text{O}+2\text{ M}{\text{V}}^{2+}\leftrightarrows {\text{CO}}_{2}+2{\text{MV}}^{+}+2{\text{H}}^{+}$$

The reaction was performed by filling a glass cuvette (Synthetic quartz glass, Hellma Analytics) with 1 mL of MV dichloride solution (pH 8) in a vinyl anaerobic chamber (Type B with incubator, Coy Laboratory Products, Inc.); each cuvette was closed with a rubber stopper and flushed with CO for 3 min (0.1 bar). Subsequently, 2–5 µL of Titanium (III) citrate solution (2 M Na₂CO₃, 0.4 M Na_3_C_6_H_5_O_7_, 15% TiCl_3_) was added, followed by 100 µL of the sample supernatant. Absorbance was measured for 30 min at 578 nm (Ultrospec 2100 pro, Amersham Biosciences).

## Supplementary Information

Supplementary material 1.

## Data Availability

No data sets were generated or analysed during the current study.
