# Supplementary material for: Batch and semi-continuous fermentation with Parageobacillus thermoglucosidasius DSM 6285 for H2 production
Source: Biotechnol Biofuels Bioprod. 2025 Jan 9;18:3. doi: 10.1186/s13068-024-02597-z (PMC11715973; doi:10.1186/s13068-024-02597-z)
Supplement: Supplementary file 1 — Supplementary material 1. [file 13068_2024_2597_MOESM1_ESM.docx]

**Additional file 1**





**Supplementary Figure 1.** Batch fermentation in mLB medium. *P. thermoglucosidasius* DSM 6285 was cultivated at 55°C, 500 rpm, pH 6.8, during 7d. The H_2_ and CO_2_ production rate (mmol min^-1^), together with the CO consumption rate (mmol min^-1^), are the average of two bioreactors, with the standard deviation indicated by the colored regions.

**Electron selectivity**

The electron fluxes were calculated based on the conversion factors and the amount of each compound, following Equation 1.

${e^{-}mmol}_{X}\left[ \frac{mol e^{-}}{d} \right]= \dot{n}_{X}*{eeq}_{X}$ (1)

Where $\dot{n}_{X}$ is the daily uptake rate of the substrates or the daily production rate of the products (mol d^-1^) and ${eeq}_{X}$ is the electron equivalents (mol e^-^/mol) for each compound.

The selectivity of the process towards the products was calculated with Equation 2.

$e^{-}mol Selectivity \left[ \% \right]= \frac{\sum{e^{-}mmol}_{Products}}{\sum{e^{-}mmol}_{Substrates}}*100\%$ (2)

The sum of the daily $e^{-}mol$ from carbon monoxide (CO) and glucose was the $\sum{e^{-}mmol}_{Substrates}$, while the sum of the daily $e^{-}mol$ from acetate, formate, lactate and propionate was presented as $\sum{e^{-}mmol}_{Products}$. However, this was decided only when consumption of the metabolites was not observed, when consumed, they were accounted as substrates in the balance.

**Table S1.** Conversion factors

| Compound | Molecular Weight (g/mol) | mol e^-^/mol |
| --- | --- | --- |
| CO | 28.0 | 2 |
| CO_2_ | 44.0 | 0 |
| Hydrogen | 2.0 | 2 |
| Glucose | 180.1 | 24 |
| Formate | 46.1 | 2 |
| Acetate | 60.0 | 8 |
| Lactate | 90.0 | 12 |
| Propionate | 74.0 | 14 |
